# Virus transmission during orthopedic surgery on patients with COVID-19 – a brief narrative review

**DOI:** 10.1080/17453674.2020.1764234

**Published:** 2020-05-14

**Authors:** Trude Basso, Håvard Dale, Håkon Langvatn, Greger Lønne, Inge Skråmm, Marianne Westberg, Tina S Wik, Eivind Witsø

**Affiliations:** a Department of Orthopedics, St Olavs University Hospital;; b Department of Orthopedics, Haukeland University Hospital;; c Faculty of Medicine, University of Bergen;; d Department of Orthopedics, Innlandet Hospital Trust;; e Faculty of Medicine and Health Sciences, Norwegian University of Science and Technology;; 6 Department of Orthopedics, Oslo University Hospital;; g Clinic of Orthopedic surgery, Akershus University Hospital, Norway

## Abstract

Background and purpose — COVID-19 is among the most impactful pandemics that the society has experienced. Orthopedic surgery involves procedures generating droplets and aerosols and there is concern amongst surgeons that otherwise rational precautionary principles are being set aside due to lack of scientific evidence and a shortage of personal protective equipment (PPE). This narrative review attempts to translate relevant knowledge into practical recommendations for healthcare workers involved in orthopedic surgery on patients with known or suspected COVID-19.

Patients and methods — We unsystematically searched in PubMed, reference lists, and the WHO’s web page for relevant publications concerning problems associated with the PPE used in perioperative practice when a patient is COVID-19 positive or suspected to be. A specific search for literature regarding COVID-19 was extended to include publications from the SARS epidemic in 2002/3.

Results — Transmission of infectious viruses from patient to surgeon during surgery is possible, but does not appear to be a considerable problem in clinical practice. Seal-leakage is a problem with surgical masks. Due to the lack of studies and reports, the possibility of transmission of SARS-CoV-2 from patient to surgeon during droplet- and aerosol-generating procedures is unknown.

Interpretation — Surgical masks should be used only in combination with a widely covering visor and when a respirator (N95, FFP2, P3) is not made available. Furthermore, basic measures to reduce shedding of droplets and aerosols during surgery and correct and consistent use of personal protective equipment is important.

Due to the COVID-19 pandemic, elective orthopedic procedures are currently, to a great extent, postponed (CDC 2020, ECDC 2020). However, patients with and without COVID-19, with cancer, infections in bones, joints, and soft tissues, critical ischemia, open and unstable fractures, and other urgent diagnoses will still be in need of orthopedic surgery.

It is widely accepted that healthcare workers (HCWs) performing procedures involving the respiratory tract face a high risk of contracting Severe Acute Respiratory Syndrome Coronavirus 2 (SARS-CoV-2). This is reflected in the WHO’s recommendation (WHO 2020b) for the highest standard of personal protective equipment (PPE) in these cases. The risk for HCWs to contract SARS-CoV-2 during orthopedic surgery, or most other surgeries for that matter, has not been studied and is consequently less clear.

Orthopedic surgery often involves the use of high-speed saws, power drills, pulsed lavage, suction, and electrocauterization. Shedding of droplets from the wound is reflected in the extensive use of protective eyewear, such as goggles and visor, in everyday practice. Concern amongst surgeons and other HCWs that otherwise rational precautionary principles are being set aside due to lack of scientific evidence and a shortage of PPE is obvious on social media platforms and amongst colleagues.

This short review is an attempt to translate relevant knowledge into practical recommendations for HCWs involved in orthopedic surgery on patients with known or suspected COVID-19.

## Transmission of SARS-CoV-2

Virus shedding through droplets that rapidly fall to the ground requires a different PPE approach to prevent transmission than infectious aerosols that can remain airborne for a longer period. According to the WHO, the transmission of SARS-CoV-2 mainly occurs through droplets (WHO 2020a). The organization concludes that transfer through aerosols is unlikely except during specific aerosol-generating procedures. As a response, Nature problematized existing controversies regarding possible air-transmission in a news story, shedding light on the complexity of the subject and that several uncertainties cannot be clarified in a long time (Anon 2020).

SARS-CoV-2 RNA (vRNA) has been found in aerosols (Ong et al. 2020, Santarpia et al. 2020). However, it is not clear whether aerosols contain infectious SARS-CoV-2, or enough viable virus to transmit the disease. We are not aware of any studies that have investigated aerosols produced during surgery on patients with SARS-CoV-2 viremia or disseminated disease.

## Presence of SARS-CoV-2 in the musculoskeletal system

SARS-CoV-2 was first identified in December 2019 and, consequently, is not fully understood. Like SARS-CoV, the coronavirus causing the 2002/3 SARS epidemic, SARS-CoV-2 binds to ACE2 receptors on human cells (Shang et al. 2020). ACE2 receptors are present on cells in the lungs and small intestines, but also on cells in a variety of other tissues, including veins, arteries, and skeletal muscle, throughout the body (Hamming et al. 2004, Riquelme et al. 2014).

Most current tests used to confirm the presence of SARS-CoV-2 use PCR technology to detect vRNA. A vRNA test will return positive with viable virus, but also with non-viable virus and virus debris. Only a viable virus can infect new individuals. To our knowledge infectious virus has been found only in respiratory tract tissue and in 2 fecal samples from 8 patients (Wang et al. 2020, Wolfel et al. 2020). We are not aware of any published studies that have aimed to find viable SARS-CoV-2 in blood, bone, bone marrow, or skeletal muscle in COVID-19 patients. An in vitro study showed replication of SARS-CoV-2 within blood-vessel organoids (Monteil et al. 2020).

Several studies have identified vRNA in blood and serum (Shang et al. 2020, Wang et al. 2020, Young et al. 2020). vRNA has been found in both the severely ill and in patients with mild symptoms. Amongst 15 patients with multiple site samples, vRNA in blood was detected in 6 patients with negative swabs from the upper respiratory system (Zhang et al. 2020).

An autopsy study including 8 deceased patients from the SARS epidemic in 2002/3 showed widespread virus dissemination in immune cells of the blood, spleen, and lymph nodes and in cells of the respiratory tract, renal tubules, intestines, and brain (Gu et al. 2005). Virus was not found within skeletal muscle cells.

## Aerosol formation during orthopedic surgery

High-speed saws, power-drills, pulsed lavage, suction, and electrocauterization are all droplet- and aerosol-generating procedures. Infected fluids, such as blood and irrigation fluid, can aerosolize during surgery and shed bacteria and viruses and have the potential to transmit disease (Heinsohn and Jewett 1993).

During experimental set-ups in vivo, infectious HIV-1 particles have been found in aerosols produced using an oscillating saw on a known infected individual (Johnson and Robinson 1991) and aerosols formed in laser fume transmitted disease in a bovine Papillomavirus model (Garden et al. 2002). Both the use of a high-speed cutter and pulsed lavage showed shedding of Staphylococcus aureus several meters from the operating field. The shedding was reduced, but not eliminated, when a drape was used as an overlying protective barrier (Nogler et al. 2001, Putzer et al. 2017). Literature is sparse, and we could not find evidence of disease transmission from patient to surgeon through aerosolized virus-infected fluids from orthopedic-like procedures in clinical practice.

## Surgical masks and particulate respirators (N95, FFP2/P3)

Originally, surgical masks were made to protect the patient from infectious pathogens in HCWs. Respirators, the somewhat confusing technical term for face masks with the standards N95, FFP2/P3, were designed to protect the user from airborne particles.

The WHO’s recommendations regarding PPE do not discuss aerosol-generating surgical procedures on infected patients (WHO 2020b). A review from the Norwegian Institute of Public Health (2020) concludes that evidence regarding the risk of aerosol transmission through aerosol-generating procedures, other than those directly or indirectly affecting the airways, is low. Respirators (N95, FFP2/P3), are consequently not recommended for open surgeries elsewhere in the body (WHO 2020b, FHI 2020).

A randomized controlled clinical trial including 446 nurses concluded non-inferiority of surgical masks when compared with N95 respirators in preventing transmission of influenza and other respiratory viruses (coronavirus included) from patients to HCWs (Loeb et al. 2009). This finding from clinical practice has been supported by 3 later meta-analyses including approximately 9,000 subjects (Smith et al. 2016, Bartoszko et al. 2020, Long et al. 2020). All the included studies were performed in non-aerosol-generating settings. N95 respirators were found to be superior to surgical masks under laboratory settings regarding filter penetration and face-seal leakage (Smith et al. 2016). Experience from the SARS epidemic stresses the importance of correct and consistent use of PPE and that this might be just as important as type of airway protection to prevent nosocomial disease transmission (Seto et al. 2003, Loeb et al. 2004). It must still be emphasized that data from the SARS outbreak in Toronto showed a trend in favor of N95 respirators over surgical masks for HCWs involved in respiratory tract procedures. The difference did not reach statistical significance, but the number of nurses included was low for this sub-analysis (n = 20, 3 infected) (Loeb et al. 2004).

## Can virus transmission occur during orthopedic surgery on patients with Covid-19?

COVID-19 is a new, harmful and rapidly spreading disease that first occurred less than 4 months ago, i.e., in December 2019. The knowledge regarding the potential of SARS-CoV-2, and the previous SARS-CoV, to spread via droplets and aerosols produced during surgery is very sparse.

Some patients with both mild and severe COVID-19 have vRNA in their blood indicative of viremia. Infectious disease transmission through both droplets and aerosols produced during orthopedic surgery is possible. In the case of SARS-CoV-2, the risk naturally depends on the virus’s capability of transmission through tissues other than respiratory tract tissues and feces. Results from possible investigations of such a capability have not been published at the time of writing (April 2020).

## Conclusions

The presence of viable virus in blood, bone marrow, or soft tissues has to our knowledge not yet been studied and should be addressed as soon as possible during this pandemic.

Our following recommendations are based on available literature at the time of writing. The absence of evidence is, in situations like this, not a sufficient reason to restrict HCWs’ access to the PPE necessary to protect against transmission of a virus with the potential to cause a severe infection.

The inferiority of surgical masks to respirators (P95, FFP2/P3) during surgery on a COVID-19 patient is not so clear. Seal-leakage due to poor fit is a problem with surgical masks. Hence, surgical masks should only be used in combination with a widely covering visor and if a respirator (P95, FFP2, P3) is not made available. Furthermore, basic protective measures should be implemented, such as avoiding pulsed lavage and the use of power tools when possible, and covering the surgical field with a waterproof drape while performing droplet- and aerosol-generating procedures. Finally, correct and consistent use of PPE is of outmost importance for orthopedic surgeons in general, and, based on current literature, probably more important than the use of respirators (P95, FFP2, P3) per se.

TB wrote the first draft in collaboration with EW. TB coordinated discussions and preparation of the final manuscript. TB, HD, GL, MW, TSW, and EW evaluated scientific papers and public recommendations, and all contributed substantially to the contents of the article.
